# A114 INTERRATER RELIABILITY BETWEEN ENDOSCOPISTS USING LOW-COST SIMULATED POLYPS

**DOI:** 10.1093/jcag/gwae059.114

**Published:** 2025-02-10

**Authors:** A Almneni, J Lisondra, N Gimpaya, A Y Zhao, D Tham, C Pattni, S Jugnundan, G S Brar, S Gupta, N Sabrie, S Grover

**Affiliations:** Royal College of Surgeons in Ireland, Dublin, Ireland; Scarborough Health Network, Scarborough, ON, Canada; Scarborough Health Network, Scarborough, ON, Canada; University of Toronto Temerty Faculty of Medicine, Toronto, ON, Canada; Scarborough Health Network, Scarborough, ON, Canada; Humber River Health, Toronto, ON, Canada; University of Toronto Temerty Faculty of Medicine, Toronto, ON, Canada; University of Toronto Temerty Faculty of Medicine, Toronto, ON, Canada; University of Toronto Temerty Faculty of Medicine, Toronto, ON, Canada; University of Toronto, Toronto, ON, Canada; Scarborough Health Network, Scarborough, ON, Canada

## Abstract

**Background:**

The identification and removal of polyps are essential skills as they remove premalignant lesions found ad hoc during colonoscopy. Simulation training helps with trainee learning curves and help train novices in polyp identification and polypectomy skills acquisition. The cost of simulators, however, limits trainee access to simulation-based training.

**Aims:**

To determine the interrater reliability between endoscopists in identifying the Paris Classification of low-cost simulated polyps.

**Methods:**

Using the Paris classification, novel simulated polyps with various morphologies were developed. 5 endoscopists of varying experience levels (1 expert (>1000 endoscopies), 2 intermediates (500-1000 endoscopies), and 2 novices (<500 endoscopies)) completed a knowledge test. The knowledge test showed images of simulated polyps and the endoscopists identified the Paris classification (Figure 1). The primary outcome measure was the interrater reliability between endoscopists during the knowledge test. A two-way random effects intraclass correlation coefficient (ICC), with absolute agreement between the raters was used to estimate the interrater reliability. Another outcome measure was content validity which was assessed via survey completed by 3 experts and 21 novice endoscopists during a previous simulation course.

**Results:**

The raters correctly identified all 1p polyps. The Average Measures ICC between all 5 raters was 0.961 (95% CI: 0.93-0.98) indicating excellent reliability. One rater correctly identified all 1s polyp and was removed from ICC analysis due to lack of variance. The Average Measures ICC for 1s polyps was 0.524 (95%CI: 0.15-0.76) indicating moderate reliability. All errors were misidentification of 1s for 2a polyps. The survey showed content validity in terms of setup, usefulness in trainee programs, realism, and perceived trainee improvement.

**Conclusions:**

Excellent reliability for 1p polyps suggests its usefulness for training polypectomy. Further work is needed to differentiate 1s polyps from 2a polyps. A limitation to interrater reliability is the use of images instead of videos in the knowledge test. The content validity of the polyps suggests practicality for low-cost training to achieve trainee improvement. Further studies are needed to assess other polyp classifications and correlate endoscopist experience level with ability to identify the simulated polyps.

Knowledge Test Scores of 5 Endoscopists

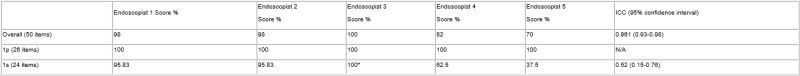

ICC, intraclass correlation coefficient. (*)excluded from ICC analysis due to lack of variance.

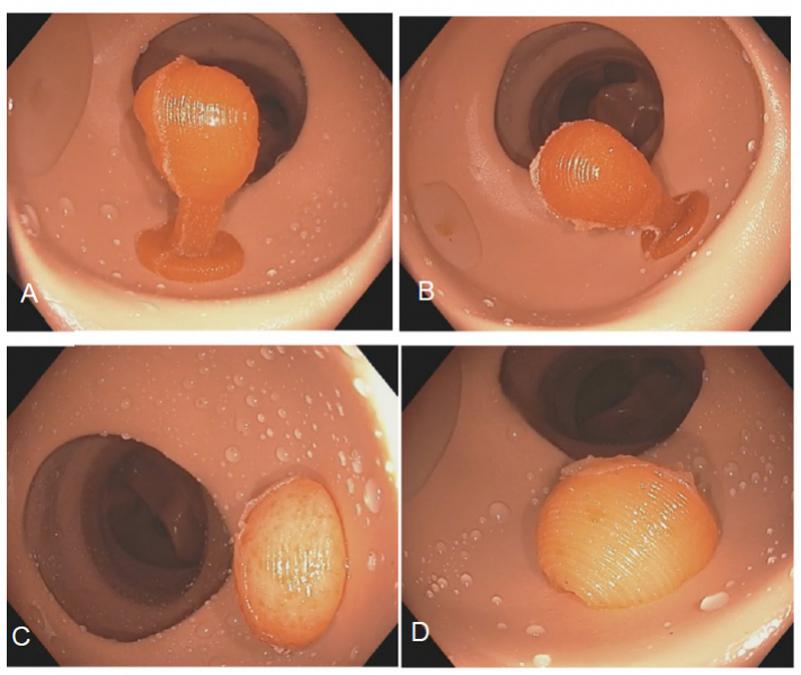

Figure 1. Simulated gel polyps based on Paris classification A-B) 1p, C-D) 1s.

**Funding Agencies:**

None

